# Episomal Viral cDNAs Identify a Reservoir That Fuels Viral Rebound after Treatment Interruption and That Contributes to Treatment Failure

**DOI:** 10.1371/journal.ppat.1001303

**Published:** 2011-02-24

**Authors:** Mark Sharkey, Dunja Z. Babic, Thomas Greenough, Roy Gulick, Daniel R. Kuritzkes, Mario Stevenson

**Affiliations:** 1 Program in Molecular Medicine, University of Massachusetts Medical School, Worcester, Massachusetts, United States of America; 2 Department of Medicine, Miller School of Medicine, University of Miami, Miami, Florida, United States of America; 3 Weill Medical College of Cornell University, New York, New York, United States of America; 4 Brigham and Women's Hospital, Harvard Medical School, Boston, Massachusetts, United State of America; Duke University Medical Center, United States of America

## Abstract

Viral reservoirs that persist in HIV-1 infected individuals on antiretroviral therapy (ART) are the major obstacle to viral eradication. The identification and definition of viral reservoirs in patients on ART is needed in order to understand viral persistence and achieve the goal of viral eradication. We examined whether analysis of episomal HIV-1 genomes provided the means to characterize virus that persists during ART and whether it could reveal the virus that contributes to treatment failure in patients on ART. For six individuals in which virus replication was highly suppressed for at least 20 months, proviral and episomal genomes present just prior to rebound were phylogenetically compared to RNA genomes of rebounding virus after therapy interruption. Episomal envelope sequences, but not proviral envelope sequences, were highly similar to sequences in rebounding virus. Since episomes are products of recent infections, the phylogenetic relationships support the conclusion that viral rebound originated from a cryptic viral reservoir. To evaluate whether the reservoir revealed by episomal sequence analysis was of clinical relevance, we examined whether episomal sequences define a viral population that contributes to virologic failure in individuals receiving the CCR5 antagonist, Vicriviroc. Episomal envelope sequences at or near baseline predicted treatment failure due to the presence of X4 or D/M (dual/mixed) viral variants. In patients that did not harbor X4 or D/M viruses, the basis for Vicriviroc treatment failure was indeterminate. Although these samples were obtained from viremic patients, the assay would be applicable to a large percentage of aviremic patients, based on previous studies. Summarily, the results support the use of episomal HIV-1 as an additional or alternative approach to traditional assays to characterize virus that is maintained during long-term, suppressive ART.

## Author Summary

Infection by HIV-1 and the related effects on human health continue to be a major problem throughout the world. Since the early 1980's, more than 25 million people have died from AIDS and the only treatment option for infected individuals is likely to be life-long treatment with a combination of antiviral drugs. While antiviral drug therapy can reduce viral replication to levels that are undetectable by currently used assays, there is a rapid recrudescence of viremia upon interruption of therapy. This indicates that there are viral reservoirs, undetectable by conventional diagnostic assays that sustain the virus in the face of ART. We have developed an alternative or additional approach to study cryptic viral replication based on episomal HIV-1 genomes. Although HIV-1 episomes are not suitable substrates for integration and thus are dead-end products in the viral life cycle, episomal HIV-1 genomes are useful surrogate markers of viral replication since they are labile and indicative of recent infection events. Here we have used episomal HIV-1 analysis to study the reservoir that fuels viral rebound during treatment interruption and to demonstrate the utility of this approach in guiding the clinical treatment of infected individuals.

## Introduction

Despite great advances in the treatment of HIV infection and disease, therapeutic eradication of viral infection is currently not possible. Individuals who respond well to ART and maximally suppress virus replication based on standard assays, harbor viral reservoirs that fuel a rapid rebound in viral replication upon interruption of antiviral treatment. Although the biological relevance of different reservoirs is subject to ongoing debate, there is evidence to support the existence of at least two distinct viral reservoirs that persist during effective, prolonged suppression of HIV-1 replication. One is a latent reservoir that is comprised of lymphocytes that were infected and subsequently reverted to a quiescent state [1]–[3]. Reactivation of latently infected cells results in viral gene expression and subsequent virion production. In patients on HAART, this reservoir has been estimated to decay with a half-life of greater than 44 months and is considered to be a major obstacle to complete clearance of HIV-1 infection [4].

In addition to latently infected cells, there is mounting evidence to support the existence of a reservoir of low-level ongoing replication in patients on HAART. Evolution of viral envelope sequences in the context of HAART has been documented for HIV-1 infected patients [5], [6] and detection of one and two long terminal repeat circles (episomal HIV-1 cDNAs) in patients on HAART indicates that new rounds of infection continue despite highly suppressive treatment [7]. While short-term *in vitro* experiments have suggested that 2-LTR circles are stable [8], [9], *in vivo* analysis has demonstrated their labile nature, which supports the use of episomal HIV-1 cDNAs as a valid surrogate marker of recently infected cells [10]–[12]. Perhaps the most compelling evidence supporting the existence of a reservoir in which low-level replication persists is derived from a study utilizing a novel antiviral drug that targets the enzymatic activity of integrase. In approximately 30% of patients on long-term suppressive antiretroviral therapy, treatment with the integrase inhibitor raltegravir led to a transient increase in 2-LTR circles and a normalization of immune activation markers [13]. These results support the conclusion that, at least in some patients, standard antiretroviral therapy is not fully suppressive and a reservoir of cryptic replication fuels viral persistence.

Other groups have also focused on defining the effects of therapy intensification on residual viremia using raltegravir, yielding different conclusions [14]–[17]. As in the study by Buzón and colleagues, one group observed an increase in 2-LTR episomal DNA levels in antiretroviral-experienced patients upon therapy intensification [15]. While a third group did not observe an increase in episomes in response to switching raltegravir for enfuvirtide [14], the effects of raltegravir were assessed at week 24 and 48, which is likely to be well after the effects of intensification occur. The remaining groups focused on measuring residual viremia in order to gauge the effects of therapy intensification using raltegravir [16], [17]. Their main conclusion is that there is no significant change in viremia in response to raltegravir treatment, so the observed residual viremia is not due to ongoing cycles of HIV-1 infection. It is unclear at this point what the residual viremia represents. If it were derived from long-lived cells that harbor integrated proviruses, one would not expect to observe an effect upon therapy intensification with a new, potent antiviral. While further work is necessary, episomal DNA may be a more accurate marker of cryptic replication than residual viremia.

In patients who suppress virus replication to levels below the limit of detection of standard assays, low-level ongoing replication can be detected through the analysis of the HIV-1 episomes. Therefore, in highly suppressed individuals who have undetectable virus in plasma, analysis via episomal HIV-1 cDNA provides the means to monitor and define the nature of actively replicating virus that persists in the face of ART.

Here we have focused on analyzing episomal envelope (*env*) sequences to probe the nature of actively replicating virus that persists during ART and demonstrate the utility of episomal sequence analysis in guiding clinical treatment of infected individuals. Envelope sequences were compared to define the phylogenetic relationships between episomal and proviral genomes prior to rebound in patients on ART to RNA genomes of virus that emerged upon therapy interruption. Additionally, episomal *env* genes were analyzed from a group of patients undergoing salvage therapy with Vicriviroc (VCV) in order to predict the virologic response to this CCR5 antagonist.

## Results

### Phylogenetic analysis of rebounding virus

**Figure 1:**
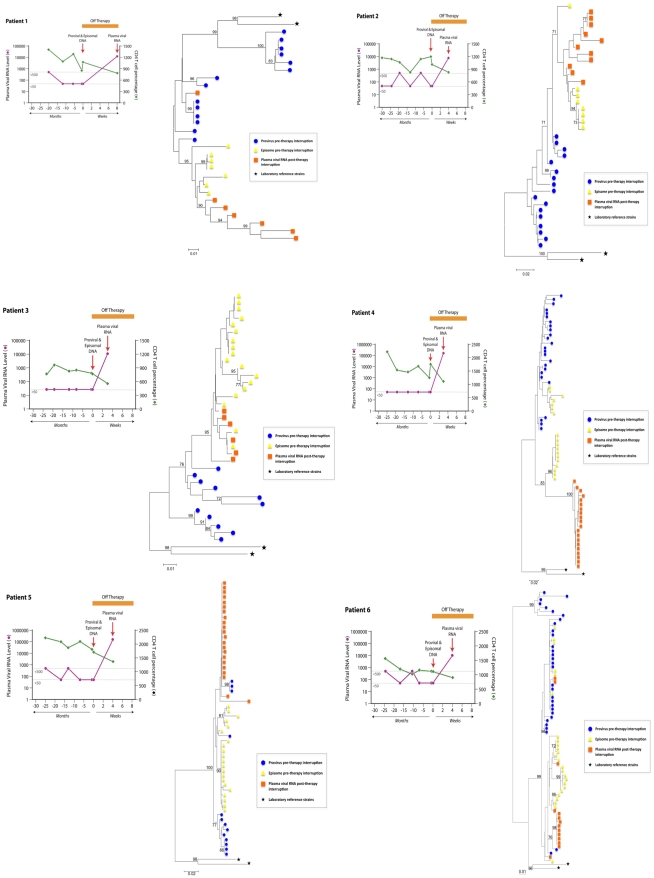
Phylogenetic trees based on the C2-V4 regions of envelope for six different patients undergoing interruption of antiviral treatment. Plasma viral RNA copies (either 500 or 50 copies/ml detection limit) and CD4^+^ T cell percentages are shown for patients prior to and after suspension of ART. In all patients, there was a rapid rebound of plasma viral RNA when therapy was discontinued. Phylogenetic relationships were estimated using the neighbor-joining method for episomal (yellow triangle) and proviral (blue circle) envelope sequences derived at therapy interruption to plasma viral RNA (orange square) envelope sequences obtained several weeks after rebound. Each symbol represents an individual sequence analyzed. Envelope sequences from HIV-1_LAI_ and HIV-1_ADA_ laboratory strains (black stars) are included for reference. Numbers at branch nodes refer to the bootstrap support; only frequencies greater than 70 percent are shown.

**Figure 2:**
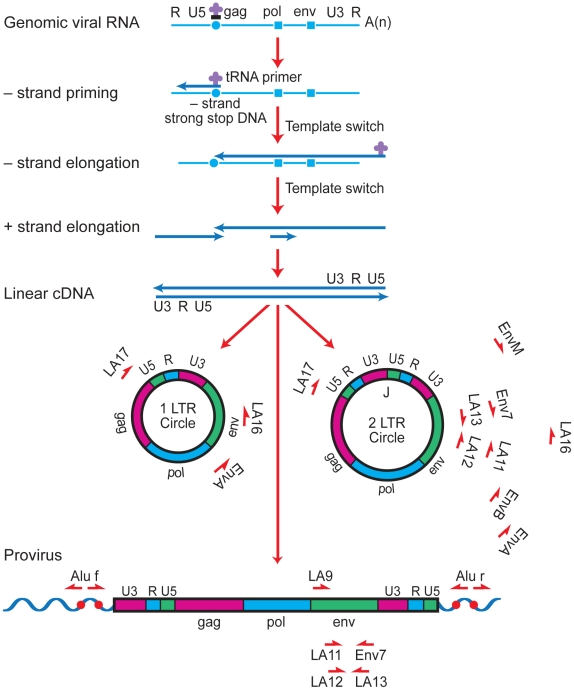
Strategy for long-range PCR amplification of episomal or proviral HIV-1 envelope sequences. Major cDNA intermediates in reverse transcription are denoted. Thin blue line, viral RNA; thick blue line, cDNA. Primer-binding sites for initiation of minus-strand cDNA synthesis and polypurine tracks for plus- or minus-strand synthesis are indicated by open circles and squares, respectively. Relative positions and orientations of first and second round PCR primers specific for the different forms of HIV-1 are listed.

Several approaches have been used in previous studies to attempt to characterize the virus that fuels the rapid rebound of viremia upon interruption of ART with conflicting results [18]–[21]. To address this issue, we compared rebound virus *env* sequences to episomal and proviral *env* sequences that were present immediately prior to treatment interruption in a group of six individuals. The rationale for this analysis is that episomal HIV-1 genomes are a dynamic marker for recent infections and are representative of a reservoir in which HIV-1 is actively replicating, while proviral genomes are predominantly archival and defective [22], [23]. Peripheral blood lymphocytes and plasma were obtained for a group of patients who had plasma viral RNA measurements below the level of detection for a minimum of 20 months (Fig 1). Episomal env sequences could not be amplified from four of the original set of patient samples and were excluded from the analysis. During the treatment period, patients suppressed viral replication with a combination of two nRTIs and at least one protease inhibitor (Table1). It should be noted that apparent increases in plasma viral RNA resulted from the use of assays with different limits of detection (<50 or <500 HIV-1 RNA copies per ml) and not from transient increases in viral replication. Upon therapy interruption, HIV-1 replication, based on plasma viral RNA measurements, rapidly resurged in all patients (Fig 1). DNA from peripheral blood lymphocytes was purified to selectively enrich for chromosomal and extrachromosomal fractions and the C2 to V4 regions of *env* were amplified by nested PCR using episomal-specific or proviral-specific primer pairs (Fig 2). Using this fractionation step greatly increases the likelihood that only the intended target DNA is amplified. The majority of unintegrated HIV-1 genomes remain in the supernatant and only a small amount of chromosomal DNA, potentially harboring an integrated provirus, partitions to the extrachromosomal fraction. Depending on the number of proviruses in a given sample, the level of contamination ranges from about 1 to 2 percent (Fig S1). Since the proviral frequency in patients on HAART is low [11], [24], amplifying *env* from a proviral template in the extrachromosomal fraction is unlikely.

**Table 1:** Profiles of the HIV-1-infected patients.

			PVL*	TCD4				
Patient ID	Date	Time point	(copies/ml)	(cells/µl)	Antiretroviral regimen			
Patient 1	06/12/97	−20 mo.	<500	1408	3TC	d4T	NFV	SQV
	01/22/98	−12 mo.	<50	1101	3TC	d4T	NFV	SQV
	07/14/98	−6 mo.	<50	1289	3TC	d4T	NFV	SQV
	12/16/98	−1 mo.	<50	863	3TC	d4T	NFV	SQV
	01/06/99	Off therapy 0 wk	<50	1078	none			
	03/05/99	Off therapy 8 wks	11466	792	none			
Patient 2	10/08/96	−30 mo.	<50	1173	3TC	d4T	IDV	
	03/04/97	−24 mo.	<50	1147	3TC	d4T	IDV	
	07/23/97	−19 mo.	<500	1066	3TC	d4T	IDV	
	01/12/98	−13 mo.	<50	832	3TC	d4T	IDV	
	07/07/98	−7 mo.	<500	1146	3TC	d4T	IDV	
	12/22/98	−1 mo.	<50	1200	3TC	d4T	IDV	
	01/13/99	Off therapy 0 wk	<50	1021	none			
	02/09/99	Off therapy 4 wks	7834	832	none			
Patient 3	09/25/97	−23 mo.	<50	765	3TC	d4T	IDV	
	01/15/98	−19 mo.	<50	962	3TC	d4T	IDV	
	07/29/98	−12 mo.	<50	825	3TC	d4T	IDV	
	11/18/98	−8 mo.	<50	852	3TC	d4T	IDV	
	06/02/99	−1 mo.	<50	781	3TC	d4T	IDV	
	06/29/99	Off therapy 0 wk	<50	767	none			
	07/22/99	Off therapy 3 wks	67033	554	none			
Patient 4	04/23/97	−25 mo.	<50	2220	3TC	AZT	IDV	
	10/08/97	−19 mo.	<50	1518	3TC	AZT	IDV	
	03/25/98	−13 mo.	<50	1454	3TC	AZT	IDV	
	09/09/98	−7 mo.	<50	1663	3TC	AZT	IDV	
	02/24/99	−1 mo.	<50	1244	3TC	AZT	IDV	
	03/23/99	Off therapy 0 wk	<50	1740	none			
	04/12/99	Off therapy 3 wks	170191	1101	none			
Patient 5	04/16/97	−25 mo.	<500	2244	3TC	d4T		
	11/24/97	−17 mo.	<50	2093	3TC	d4T		
	03/16/98	−13 mo.	<500	1863	3TC	d4T	IDV	
	8/31/98	−7 mo.	<50	2106	3TC	d4T	IDV	
	02/16/99	−1 mo.	<50	1819	3TC	d4T	IDV	
	03/09/99	Off therapy 0 wk	<50	1720	none			
	04/05/99	Off therapy 4 wks	44235	1368	none			
Patient 6	03/31/97	−24 mo.	<500	1550	3TC	d4T		IDV
	10/15/97	−17 mo.	<50	1187	3TC	d4T	AZT	IDV
	03/30/98	−11 mo.	<50	1012	3TC		AZT	IDV
	07/28/98	−7 mo.	<500	1149	3TC		AZT	IDV
	01/04/99	−1 mo.	<50	1138	3TC		AZT	IDV
	01/26/99	Off therapy 0 wk	<50	1117	none			
	02/23/99	Off therapy 4 wks	10684	892	none			

PVL, plasma viral load; TCD4, total CD4 cells; 3TC, lamivudine; d4T, stavudine; AZT, zidovudine; NFV, nelfinavir; SQV, saquinavir; IDV, indinavir.

*Plasma viral loads were measured using assays that have a limit of detection of either 50 or 500 copies/ml.

In order to promote phylogenetic comparison between baseline and rebounding viral genomes, plasma virus was sampled shortly after viral rebound. This minimized sequence diversity that develops during uncontrolled virus replication at therapy interruption. *Env* sequences from plasma virions collected from plasma sampled shortly after rebound were generated by RT-PCR. Nucleotide sequences were aligned using ClustalW (MacVector 8.1) and neighbor-joining phylogenetic trees were constructed using the Kimura two-parameter model as implemented in the MEGA Version 4 program [25]. Confidence values for individual branches using 1000 replicates were estimated by bootstrap re-sampling of the neighbor-joining trees (Fig 1). Since the initial phylogenetic analyses may have included sequences based on PCR re-sampling, redundant sequences were removed from the alignments and best trees were generated using MacVector 8.1 to demonstrate data consistency and calculate genetic distances for different sequences (Figs S2–S7). All patient sequences were aligned to each other and in each case the patient sequences clustered with one another, but not with sequences from other patients or reference lab strains. In the majority of patients, *env* sequences in the rebounding virus were highly similar to episomal sequences present just prior to treatment interruption, but not to proviral sequences obtained from the same sampling time (Fig 1, Figs S2–S7). In some patients, there was tight intermingling of rebound RNA and episomal sequences demonstrating very close genetic similarity supporting the conclusion that viral rebound originated from a reservoir in which viral replication was ongoing (Fig 1, Figs S2–S4).

### Predicting viral tropism from HIV-1 episomes

Phylogenetic analysis of viral *env* sequences in individuals who interrupted therapy indicated that plasma viremia originated from a cryptic viral reservoir as identified by episomal sequences (Fig 1). However, it is possible that the viral variants identified by episomal sequences were of limited biological relevance to the immunopathogenic status of the host. To evaluate whether the cryptic viral reservoir that is revealed by episomal viral cDNA was of virologic relevance, we examined whether episomal sequences define a viral population that contributes to the virologic outcome of individuals receiving co-receptor inhibitors. HIV-1 infected individuals most frequently fail CCR5 inhibitor therapy because of the presence of a low frequency of X4 virus variants that are insensitive to CCR5 antagonists. Therefore, we hypothesized that identification of X4 viruses based on V3 *env* sequences in episomal cDNA would predict treatment failure. From ACTG trial 5211 in which patients received the CCR5 antagonist Vicriviroc (VCV), we selected fourteen patients who experienced protocol-defined virologic failure. Virologic failure for this protocol is defined as failure to experience a log reduction in viral replication from baseline values at or after week 16. Episomal *env* sequences were generated by PCR from lymphocyte DNA isolated from baseline through week 48 after initiation of VCV therapy. Full-length *env* genes were amplified from episomal cDNA and cloned into an expression vector. Pseudotyped viruses were generated with patient-derived envelopes and an NL4-3 proviral clone expressing luciferase in place of envelope. Co-receptor dependence was then gauged in an infection assay utilizing U87-based indicator cells. Amplification of full-length *env* genes was problematic for a small number of samples. Therefore, in order to derive secondary information on virus genotypes from these samples, short fragments spanning the V3 region of *env* were amplified and sequenced. Tropism predictions were determined using the Geno2Pheno algorithm with a false positive rate of five and ten percent as well as coupled 11/25 and net charge rules. The phenotypic/genotypic data is summarized in Tables 2 and S1 and tropism assignments based on an RNA phenotyping assay (Trofile, Monogram Biosciences) are listed for comparison. We were able to amplify either full-length or short episomal *env* fragments from 74 of 91 (81.3%) PBMC samples. The Trofile assay and phenotypic assay based on episomal *env* sequences were concordant at all timepoints for 8 of the 14 patients. In these individuals, R5 viruses persisted at all time points based on both phenotypic assays and virologic failure could not be explained by outgrowth of X4 virus (Tables 2, S1). In the remaining 6 patient samples, tropism assignments resulted in notable discordance. In one individual (Patient A), the Trofile assay indicated the presence of D/M virus at all time points, while our assay detected only CCR5-dependent virus. PBMC samples from patients J and K showed the emergence of X4 or D/M viruses at baseline or week 2, which was prior to assignments based on the Trofile assay. However, co-receptor use based on re-testing of baseline samples with an enhanced-sensitivity Trofile assay revealed the presence of D/M viral variants in patients D, J, and K, which is in accordance with our assay based on episomal *env* sequences [26,Table S2]. Therefore, analysis of tropism from sequences contained with *env* of episomal cDNA revealed the presence of X4 variants at or near baseline and predicted virologic failure.

**Table 2:** Tropism assignments based on HIV-1 *env* sequences derived from episomal cDNA and plasma viral RNA.

Patient ID and time point (# of clones)	log _10_ HIV-1 RNA copies/ml	Amplification of *env* gene from episomes	Phenotype Genotype* (episomal cDNA)	Trofile assay (plasma viral RNA)
*Patient A*				
baseline	4.6	no sample	-	DM
week 2 (5)	3.8	full-length	R5	DM
week 4 (3)	4.5	V3	R5*	-
week 8 (4)	4.1	full-length	R5	DM
week 16 (4)	4.2	full-length	R5	-
week 24 (5)	3.3	full-length	R5	DM
week 48	1.2	NA	-	-
*Patient B* a				
baseline (5)	5.3	full-length	R5	R5
week 2 (5)	5.8	full-length	R5	R5
week 4 (5)	5.6	full-length	R5*	-
week 8 (5)	5.5	full-length	R5	R5
week 16 (5)	4.7	full-length	R5	-
week 24 (5)	5.2	full-length	R5	R5
week 48 (1)	6.5	full-length	R5*	R5
*Patient D*				
baseline	5.6	no sample	-	R5
week 2 (5)	6.2	full-length	D	R5
week 4 (5)	5.5	full-length	D	-
week 8 (5)	5.0	full-length	R5	R5
week 16 (5)	4.6	full-length	R5	-
week 24 (1)	4.6	full-length	R5	-
week 46 (5)	4.1	full-length	D	R5
*Patient G*				
baseline	5.2	no sample	-	R5
week 2	4.9	NA	-	R5
week 4	4.6	NA	-	-
week 8 (2)	5.2	full-length	X4 R5	DM
week 16 (5)	4.3	V3	R5*	-
week 48 (3)	5.1	full-length	R5*	DM
*Patient J*				
baseline (5)	4.5	full-length	X4	R5
week 2 (4)	4.6	full-length	X4 R5	R5
week 4 (3)	4.3	full-length	X4	-
week 8 (2)	4.2	full-length	X4 R5	X4
week 16 (1)	4.3	full-length	X4	-
week 24	4.5	NA	-	DM
week 48	5.1	NA	-	DM
*Patient K*				
baseline	4.5	no sample	-	R5
week 2 (5)	4.2	full-length	D R5	R5
week 4 (5)	4.6	full-length	R5	-
week 8 (4)	4.5	full-length	D R5	R5
week 16 (5)	4.2	V3	X4 R5*	-
week 26 (5)	4.6	V3	X4 R5*	-
week 48 (4)	4.4	V3	R5*	DM
*Patient L*				
baseline	4.6	no sample	-	DM
week 2 (5)	5.0	full-length	D R5	R5
week 4 (1)	5.5	full-length	R5	-
week 8 (2)	5.8	full-length	X4 R5*	R5
week 16 (5)	5.5	V3	R5*	-
week 48	1.2	NA	-	-

NA, no amplification of full-length or short *env* fragment; DM, dual/mixed; D, dual-tropic virus.

*If amplification of the full-length *env* gene was not successful or if the phenotypic assay failed, secondary information on the virus genotype was derived by amplifying and sequencing a short fragment spanning the V3 region of *env*.

a Seven more patients with concordant results between the two assays are presented in Table S1.

## Discussion

Although current antiviral treatment can successfully reduce viral loads to undetectable levels in HIV-1 infected individuals, viral reservoirs persist. In order to guide efforts aimed at eradication of the virus from infected cells, it is critical to develop a comprehensive knowledge of the viral reservoirs that are maintained in the face of suppressive antiviral treatment. Since well-suppressed individuals have extremely low to undetectable levels of virus in peripheral blood, an alternative to virion RNA analysis is needed. Detection and analysis of episomal HIV-1 genomes, in patients in which virus replication is suppressed to levels below the limit of detection of standard assays, provides an option to characterize virus that persists in such individuals. We have previously shown that episomes are labile *in vivo*, and therefore, are valid surrogate markers for recent infections [10].

Here we have applied episomal analysis to characterize virus that rebounds upon antiviral treatment interruption. In an analysis of six patients, there was a rapid rebound in plasma viral RNA when therapy was interrupted after a prolonged period of sustained suppression of viral replication. Phylogenetic relationships based on C2 to V4 *env* sequences indicated that in the majority of patients, rebounding virus was highly similar to episomal sequences that existed prior to treatment interruption, but were not closely related to proviral sequences present at the same sampling time. As such, HIV-1 episomal cDNA is representative of a relevant biological reservoir that persists in individuals on ART and whose characteristics may not be defined by other assays. We observed considerable variability in the diversity of episomal sequences derived from the different patients. In patients on HAART, episomal HIV-1 genomes are rare and inter-patient variability can be high. We have previously observed that 2-LTR HIV-1 quantities in patients receiving highly suppressive antiviral therapy range from less than one to greater than 600 copies per million PBMCs [7]. In this study, *env* sequence diversity was dependant on the relative abundance of episomal genomes present in the samples (data not shown).

Previous studies aimed at revealing the origin of virus that rebounds upon therapy interruption have reached different conclusions. Both activation of latently infected CD4^+^ T cells and low-level active viral replication have been implicated in the rapid resurgence of viremia following treatment interruption [18]–[21], [27]. Our analysis provides additional *in vivo* evidence that residual viral replication persists in patients on highly suppressive therapies and that viral rebound is fueled by this cryptic replication. While the present study supports the hypothesis that HIV-1 rebounds from a reservoir in which replication is ongoing, a recent study by Joos et al. concluded that rebounding virus is derived from the activation of latently infected cells [27]. The conclusion is based upon comparisons of residual viremia and virus that rebounds upon multiple treatment interruptions. Although the data is consistent with activation from latently infected cells, analysis of proviruses was not included, so one cannot be certain of the source of rebounding virus. Here we have directly compared proviruses and episomes to rebounding virus and conclude that it is more likely that the rebounding virus is derived from actively replicating virus. Work by others has suggested that at least some of the plasma virions that are produced in the setting of ART harbor replication competent virus [28]. Although this suggests that residual viremia can fuel HIV-1 rebound upon therapy interruption, the study was done with a single patient, so it is difficult to generalize the findings. Phylogenetic analysis of tat sequences derived from plasma virions and CD4^+^ T cells from the same patient revealed that most sequences generated from plasma were distinct from those in CD4^+^ T cells, which is consistent with previous studies [29]–[31]. These studies have concluded that residual viremia is derived from a source other than CD4^+^ T cells. Our analysis focused on genomes that were associated with PBLs, and given the genetic similarity between episomes and rebounding virus, episomal genomes may be derived from a reservoir that is distinct from the source of residual viremia. Although it would be potentially informative to compare *env* sequences that we generated to *env* present in patient plasma prior to rebound, it was not possible due to the archival nature of our samples. Additionally, one might predict that the sources of residual viremia and episomes are distinct based on the results of therapy intensification with raltegravir in which episomes increase transiently [13], [15] while residual viremia remains unchanged [16], [17]. Evidently, more work is needed to better define the viral reservoirs that persist despite highly suppressive ART.

To further support the methodology and demonstrate that episomal analysis has the potential to provide information with which to guide clinical decisions regarding patient therapy, we analyzed samples from individuals undergoing salvage treatment with the CCR5 antagonist VCV. Although these patients had varying levels of viremia, we wanted to determine whether episomal HIV-1 cDNA analysis could provide information on the effects of adding VCV to a regimen that was no longer effective in suppressing viral replication. We were able to generate full-length *env* genes from sixty-four percent of the samples and of the samples in which viral plasma RNA copies were below 100, we could amplify *env* fifty percent of the time. Thus, targeting episomal genomes provides a feasible approach to the study of residual virus replication.

Although VCV is considered to be a potent inhibitor of R5 HIV-1 *in vitro* and *in vivo* [32], [33], the patients we focused on exhibited a poor response to therapy as defined by the clinical protocol. In five patients, phenotypic or genotypic analysis of episomal *env* genes indicated the presence of dual or X4 viruses while undergoing therapy. For these patients, it is highly likely that failure to respond to VCV therapy was due to the presence of viral variants that were unaffected by the CCR5 antagonist. In all cases, D/M or X4 viruses were present at early time points of the trial, so it is unlikely that selective pressure from VCV therapy resulted in a replacement of R5 with X4 virus. We observed very little variability in the primary V3 loop sequences of the patient samples, so the basis for VCV therapy failure is unclear in the subset of patients that did not harbor X4 or D/M viruses. These results are consistent with previous work studying the same group of patients [34] and imply that resistance to the antagonist may involve *env* sequences that are peripheral to the V3 loop or involve other, as yet undefined determinants. Alternatively, patients who experienced virologic failure with R5 virus may not have been fully compliant with the regimen.

Since most of the samples were analyzed while patients were experiencing viremic episodes, we were able to compare our assay data to phenotypic data previously obtained via the Trofile assay [33]. Trofile is a single-cycle recombinant virus assay in which pseudoviruses are generated from full-length RNA-derived *env* genes to determine HIV-1 tropism from plasma virions [35]. This assay is the current “gold standard” to determine viral tropism and to guide whether CCR5 antagonists should be considered in the clinical management of an infected individual. Clinicians need tropism determinations to exclude the possibility that CCR5 antagonist use will result in outgrowth of a more pathogenic virus utilizing the CXCR4 co-receptor. However, a significant limitation to the assay is the requirement that viral plasma RNA levels be greater than 1000 copies/ml [35]. The Trofile assay and phenotypic assay based on episomal *env* sequences were highly concordant. The high degree of correlation between the two assays indicates that episomes are not archival, but are evolving and derived from a reservoir of actively replicating virus. While the majority of samples analyzed were from time points in which viral plasma RNA was greater than the limit of detection, we would expect episomal analysis to be a viable approach in patients that suppress virus replication to levels below the limit of detection of standard assays. Having a method to determine viral co-receptor usage in patients with highly suppressed viremia would be advantageous for patient management. It is common for patients to experience toxicity from the drug regimens in use and defining the virus co-receptor dependence would allow a clinician to switch therapy to a better tolerated drug such as a CCR5 antagonist.

Previous studies have shown that 2- LTR episomes are detectable in a high percentage of patients on long-term highly suppressive antiviral therapy [7], [13]. Additionally, the approach we used to amplify *env* from episomes also targets 1-LTR circles that are approximately ten times more abundant than 2-LTR circles resulting in a much greater sensitivity of detection [36]. Taken together, the results support the use of episomal HIV-1 cDNA as an additional or alternative approach to traditional assays to monitor the effects of antiviral therapy, and to help guide decisions on which patients may be most responsive to co-receptor inhibitor therapy, even for patients in which viremia is undetectable based on standard assays.

## Materials and Methods

### Ethics statement

After obtaining written informed consent, cells and plasma were collected by phlebotomy from HIV-1 infected volunteers at the NIAID and research centers involved in AIDS Clinical Trials Group (ACTG) protocol A5211. Eligible participants were age 18 or older and either voluntarily suspended ART or chose to receive a new antiviral compound in the context of salvage therapy. The study was reviewed and approved by both the NIAID and the University of Massachusetts's institutional review boards. Human experimentation guidelines of the US Department of Health and Human Services were followed in the conduct of this research.

### Rebound study

#### Patients

Six HIV-1 infected individuals who were receiving effective antiviral treatment for approximately 2 years and subsequently suspended therapy were studied. None of the patients exhibited any detectable viremia prior to suspension of therapy, based on plasma viral RNA measurements (limit of detection 50 or 500 copies per milliliter). PBMCs were obtained prior to, and at the time of, therapy interruption via leukapheresis by Ficoll-Hypaque density gradient centrifugation. One milliliter of plasma was obtained 3, 4 or 8 weeks after suspension of therapy at which time viral rebound had occurred. The study was approved by the NIAID's institutional review board and informed consent was obtained in writing from study participants. Human experimentation guidelines of the US Department of Health and Human Services were followed in the conduct of this research.

#### DNA and RNA purifications

Low molecular weight DNA was purified using a modified plasmid isolation procedure (Qiagen) and chromosomal DNA was subsequently recovered from the SDS-precipitate by extraction with DNazol (Invitrogen). Cryopreserved PBMCs were collected by centrifugation at 4 KRPM for 5 min and DNA was purified according to the manufacturer's modified plasmid protocol for low copy number samples. SDS-precipitates were then thoroughly resuspended in 900 µL DNazol at 50°C and DNA was precipitated by the addition of 500 µL 100% ethanol. DNA was pelleted by centrifugation at 12 KRPM for 8 min, washed in 1.4 ml 70% ethanol and resuspended in 50 µL 8 mM NaOH. After resuspension, lysates were neutralized by the addition of 50 µL 10 mM Tris-HCl, pH 8.0. Virions were pelleted from thawed plasma at 14 KRPM for 2 h at 4°C and RNA was purified using an RNAeasy kit according to the manufacturer's protocol (Qiagen) and eluted in 50 µL water.

#### Amplification and sequencing of env genes

A portion of the envelope gene spanning the C2 to V4 region was amplified by nested PCR using limiting dilution replicates of the first round products. First round products were generated using episomal-specific (LA16/f, T^5905^ GGGTCACAGTCTATTATGGGGTA and LA17/r, T^332^ CTCCTTCTAGCCTCCGCTAGTCAA) or proviral-specific primers (Alu/f, CTCACGCCTGTAATCCCAGCA or Alu/r, TGCTGGGATTACAGGCGTGAG) and LA9/f, A^6015^ CATGCCTGTGTACCCACAGA) and Jumpstart RedAccuTaq (Sigma) in a reaction containing 1X buffer, 250 µM dNTPs, 0.5 µM primers and 1.5 units polymerase. PCR conditions consisted of a denaturation step at 95°C for 1 min followed by 22 cycles of 15 sec at 95°C, 30 sec at 58°C and 5 min at 68°C. Second round products were generated using 40 cycles of amplification as above except with an extension time of 1 min and LA11/f (C^6546^ ACAGTACAATGTACACATGGA) and Env7/r (A^7128^ GGGGCATACATTGCTTTTCCTA) envelope primers. 8 µL viral RNA was converted to cDNA using Superscript RT (Invitrogen) according to the manufacturer's protocol. cDNA was amplified by limiting dilution pcr using the same conditions as the second round reactions above. PCR products were purified and used directly for sequencing with the LA11/f primer. Primers were numbered according to the HIV-1_LAI_ consensus (K02013).

#### Phylogenetic analysis

Nucleotide sequences corresponding to the C2 to V4 regions of *env* were edited and aligned using ClustalW (MacVector 8.1) and phylogenetic relationships were generated using the neighbor-joining method included in the MEGA 4 software package with a Kimura-2 parameters distance matrix. Confidence limits for individual branches were estimated by bootstrap re-sampling of the neighbor-joining trees (1000 replicates).

### Co-receptor study

#### Patients and samples

The study was conducted using archival patient samples that had originally been collected under AIDS Clinical Trials Group (ACTG) protocol A5211, a phase 2b study of the investigational CCR5 antagonist Vicriviroc (VCV). The study was approved by the University of Massachusetts's institutional review board and informed consent was obtained in writing from study participants. Human experimentation guidelines of the US Department of Health and Human Services were followed in the conduct of this research. Ninety-one cryopreserved PBMCs were obtained from fourteen HIV-1-infected, treatment-experienced patients, who experienced protocol-defined virologic failure. PBMCs were collected during the period from study entry through week 48 (week 0, 2, 4, 8, 16, 24 and 48). PBMCs at study entry (week 0) were available only for six out of fourteen patients.

#### DNA purifications

Nucleic acid extractions were performed from patients' cryopreserved PBMCs samples (5×10^6^ cells). PBMCs were collected by centrifugation at 4 KRPM for 5 min. Cell pellets were resuspended in buffer P1 and extrachromosomal DNA was purified using a QIAprep spin miniprep kit (Qiagen) using the modification for the isolation of low copy number plasmids as recommended by the manufacturer. Final elution of the purified DNA was in 120 µl of elution buffer preheated at 70°C.

#### Amplification and cloning of full-length envelopes

HIV-1 full-length *env* sequences within episomal DNA (1- and 2-LTR) were amplified from 20–30 µl of extrachromosomal DNA by nested PCR using the Phusion Hot Start High-Fidelity DNA Polymerase (Finnzymes, Espoo, Finland). Amplifications were done in a 50-µl reaction containing 1 x Phusion HF Buffer, 200 µM deoxynucleotide triphosphates, 0.5 µM primers, and 1 U of Phusion Hot Start DNA Polymerase. Outer primers were envA (forward, T^5434^AGAGCCCTGGAAGCATCCAGGAAG) and LA17 (reverse, T^332^CTCCTTCTAGCCTCCGCTAGTCAA), and inner primers were envB (forward, CACCT^5539^AGGCATCTCCTATGGCAGGA) and envM (reverse, C^8501^CAGGTCTCGAGATGCTGCTCCCA). Primers were numbered according to the HIV-1_LAI_ consensus (K02013).

After an initial denaturation at 98°C for 1 min, first round products were generated by cycling 25 times at 98°C for 15 s, 60°C for 30 s, and 72°C for 3.30 min, followed by a final extension at 72°C for 5 min. Two microliters from the first round were then used for nested PCR which was performed with an initial denaturation step of 98°C for 1 min, followed by 25–30 cycles of 98°C for 15 s, 63°C for 30 s, and 72°C for 1.45 min, and a final extension of 72°C for 5 min.

PCR product DNA was purified over a column using QIAquick PCR Product Purification Kit (Qiagen) and cloned into the pcDNA3.1D/V5-His-TOPO expression vector (pcDNA3.1 Directional TOPO Expression Kit, Invitrogen). Plasmids were isolated with Qiagen miniprep purification kit (Qiagen) and were digested with HindIII and XhoI to confirm insertion of full-length *env*.

#### Amplification and cloning of partial env sequences

Short *env* fragment, encompassing V3 region was amplified within episomal cDNA by nested PCR using envA/LA17 as outer primers and LA12 (forward, A^6602^ TGGCAGTCTAGCAGAAGAAGA) and LA13 (reverse, T^6927^ TCTGGGTCCCCTCCTGAGGA) as inner primers. The PCR products were subjected to clonal analysis using StrataClone Blunt PCR Cloning Kit (Stratagene).

#### Genotypic characterization of HIV-1 co-receptor usage

The V3 region was sequenced from cloned *env* with LA12 primer using an ABI/Prism 377 DNA sequencer (Applied Biosystems). Sequences were edited and aligned with MacVector software (v.10.0.2). To predict HIV-1 tropism from the V3 genotype we used bioinformatic tool Geno2pheno (http://coreceptor.bioinf.mpi-sb.mpg.de/cgi-bin/coreceptor.pl) with a false positive rate of 5% and 10%, as well as, coupled 11/25 and net charge rules.

#### Phenotypic characterization of HIV-1 co-receptor usage

The phenotype of HIV-1 co-receptor usage was determined using a single-cycle recombinant virus entry assay. Human embryonic kidney 293T cells were co-transfected using Lipofectamine 2000 (Invitrogen) with pNL43-Δenv-Luc vector DNA and expressing vector containing the amplified full-length *env* sequence. Virus particles released into the supernatant after 48 hours were used to infect indicator cells bearing CD4 and CCR5 (U87.CD4.CCR5 cells) or CD4 and CXCR4 (U87.CD4.CXCR4 cells) (these reagents were obtained through the NIH AIDS Research and Reference Reagent Program, Division of AIDS, NIAID, NIH). Recombinant viruses with known co-receptor usage (X4 LAI, R5 LAI and D/M 89.6 virus) were used as controls in each assay. 293T cells transfected with empty vector plasmid were used as negative control. The ability of pseudoviruses to infect target cells was assessed by measuring the luciferase activity in lysed cells (as relative light units; RLU) 72 h postinoculation with Bright-Glo Luciferase Assay System (Promega). Tested virus was assigned a particular tropism if infection of cells expressing the relevant co-receptor resulted in an RLU reading above the background. The specificity of low-level positive luminescent signals was confirmed using co-receptor antagonists. Bicyclam JM-2987 (hydrobromide salt of AMD-3100) was used to block CXCR4-mediated entry and TAK-779 to block CCR5-mediated entry (these reagents were obtained through the NIH AIDS Research and Reference Reagent Program, Division of AIDS, NIAID, NIH). We used three concentrations (1∶3 dilutions) for each component, starting at 1.25 µg/ml for AMD-3100 and 1.5 µg/ml for TAK-779.

## Supporting Information

Figure S1 Fractionation of PBL DNA results in minimal proviral contamination of extrachromosomal supernatants. Variable numbers of 8E5 cells, which have a single HIV-1 _LAI_ provirus per cell, were combined with a constant 8×10^6^ uninfected PBLs. DNA was purified as is described in the methods section and quantitative PCR was used to measure the number of proviruses that partitioned to the extrachromosomal fraction.
(3.00 MB TIF)

Figure S2 Phylogenetic tree based on the C2-V4 regions of envelope for patient 1 undergoing interruption of antiviral treatment. Phylogenetic relationships were estimated using the neighbor-joining method to generate best tree with genetic distances for episomal (yellow triangle) and proviral (blue circle) envelope sequences derived at therapy interruption to plasma viral RNA (orange square) envelope sequences obtained several weeks after rebound. Envelope sequences from HIV-1_LAI_ and HIV-1_ADA_ laboratory strains (black stars) are included for reference.
(0.21 MB TIF)

Figure S3 Phylogenetic tree based on the C2-V4 regions of envelope for patient 2 undergoing interruption of antiviral treatment. Phylogenetic relationships were estimated using the neighbor-joining method to generate best tree with genetic distances for episomal (yellow triangle) and proviral (blue circle) envelope sequences derived at therapy interruption to plasma viral RNA (orange square) envelope sequences obtained several weeks after rebound. Envelope sequences from HIV-1_LAI_ and HIV-1_ADA_ laboratory strains (black stars) are included for reference.
(0.32 MB TIF)

Figure S4 Phylogenetic tree based on the C2-V4 regions of envelope for patient 3 undergoing interruption of antiviral treatment. Phylogenetic relationships were estimated using the neighbor-joining method to generate best tree with genetic distances for episomal (yellow triangle) and proviral (blue circle) envelope sequences derived at therapy interruption to plasma viral RNA (orange square) envelope sequences obtained several weeks after rebound. Envelope sequences from HIV-1_LAI_ and HIV-1_ADA_ laboratory strains (black stars) are included for reference.
(0.34 MB TIF)

Figure S5 Phylogenetic tree based on the C2-V4 regions of envelope for patient 4 undergoing interruption of antiviral treatment. Phylogenetic relationships were estimated using the neighbor-joining method to generate best tree with genetic distances for episomal (yellow triangle) and proviral (blue circle) envelope sequences derived at therapy interruption to plasma viral RNA (orange square) envelope sequences obtained several weeks after rebound. Envelope sequences from HIV-1_LAI_ and HIV-1_ADA_ laboratory strains (black stars) are included for reference.
(0.31 MB TIF)

Figure S6 Phylogenetic tree based on the C2-V4 regions of envelope for patient 5 undergoing interruption of antiviral treatment. Phylogenetic relationships were estimated using the neighbor-joining method to generate best tree with genetic distances for episomal (yellow triangle) and proviral (blue circle) envelope sequences derived at therapy interruption to plasma viral RNA (orange square) envelope sequences obtained several weeks after rebound. Envelope sequences from HIV-1_LAI_ and HIV-1_ADA_ laboratory strains (black stars) are included for reference.
(0.21 MB TIF)

Figure S7 Phylogenetic tree based on the C2-V4 regions of envelope for patient 6 undergoing interruption of antiviral treatment. Phylogenetic relationships were estimated using the neighbor-joining method to generate best tree with genetic distances for episomal (yellow triangle) and proviral (blue circle) envelope sequences derived at therapy interruption to plasma viral RNA (orange square) envelope sequences obtained several weeks after rebound. Envelope sequences from HIV-1_LAI_ and HIV-1_ADA_ laboratory strains (black stars) are included for reference.
(0.26 MB TIF)

Table S1 Tropism assignments based on HIV-1 env sequences derived from episomal cDNA and plasma viral RNA from patients with concordant results between the two assays.
(0.08 MB DOC)

Table S2 Co-receptor use at baseline as determined by the enhanced-sensitivity Trofile assay.
(0.04 MB DOC)
