# Supplementary figures and images for: Episomal Viral cDNAs Identify a Reservoir That Fuels Viral Rebound after Treatment Interruption and That Contributes to Treatment Failure
Source: PLoS Pathog. 2011 Feb 24;7(2):e1001303. doi: 10.1371/journal.ppat.1001303 (PMC3044693; doi:10.1371/journal.ppat.1001303)

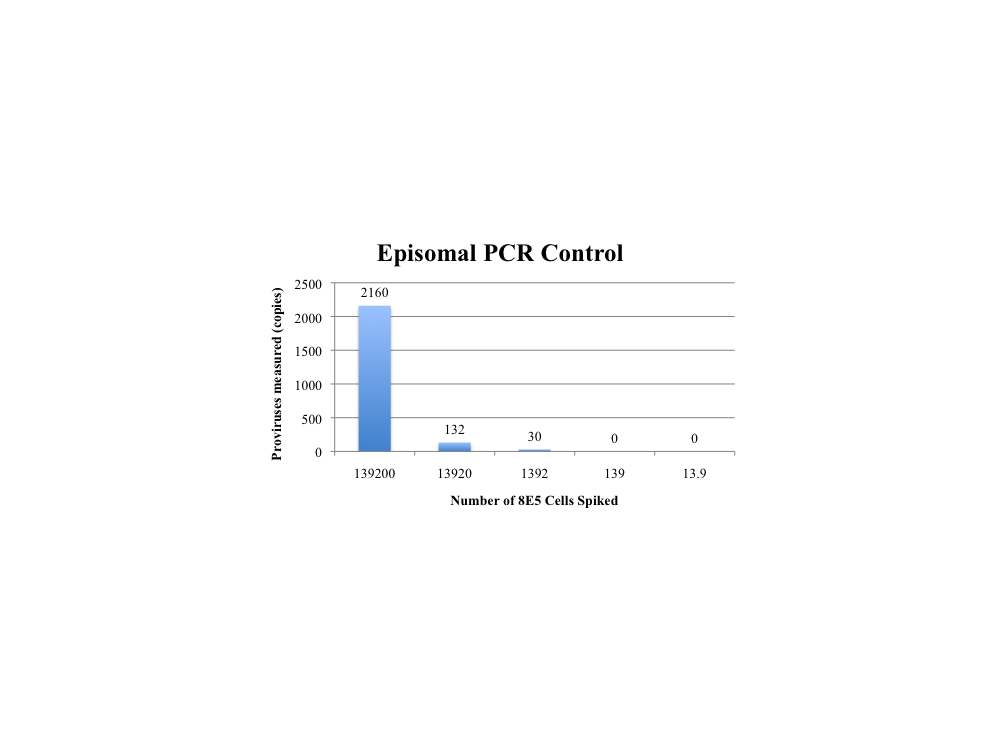

Supplement: Figure S1 — Fractionation of PBL DNA results in minimal proviral contamination of extrachromosomal supernatants. Variable numbers of 8E5 cells, which have a single HIV-1 LAI provirus per cell, were combined with a constant 8×10^6^ uninfected PBLs. DNA was purified as is described in the methods section and quantitative PCR was used to measure the number of proviruses that partitioned to the extrachromosomal fraction. (3.00 MB TIF) [file ppat.1001303.s001.tif]

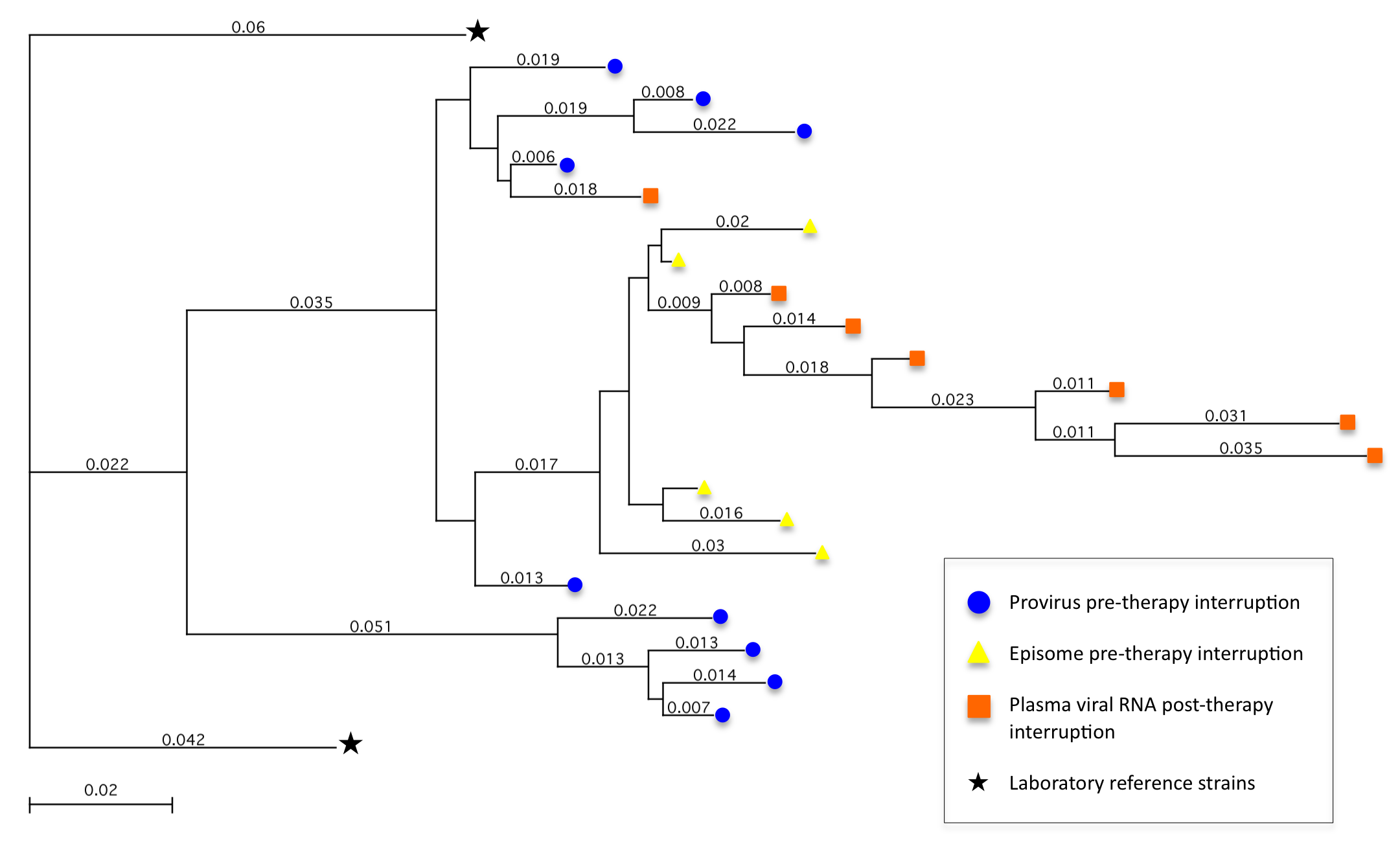

Supplement: Figure S2 — Phylogenetic tree based on the C2-V4 regions of envelope for patient 1 undergoing interruption of antiviral treatment. Phylogenetic relationships were estimated using the neighbor-joining method to generate best tree with genetic distances for episomal (yellow triangle) and proviral (blue circle) envelope sequences derived at therapy interruption to plasma viral RNA (orange square) envelope sequences obtained several weeks after rebound. Envelope sequences from HIV-1LAI and HIV-1ADA laboratory strains (black stars) are included for reference. (0.21 MB TIF) [file ppat.1001303.s002.tif]

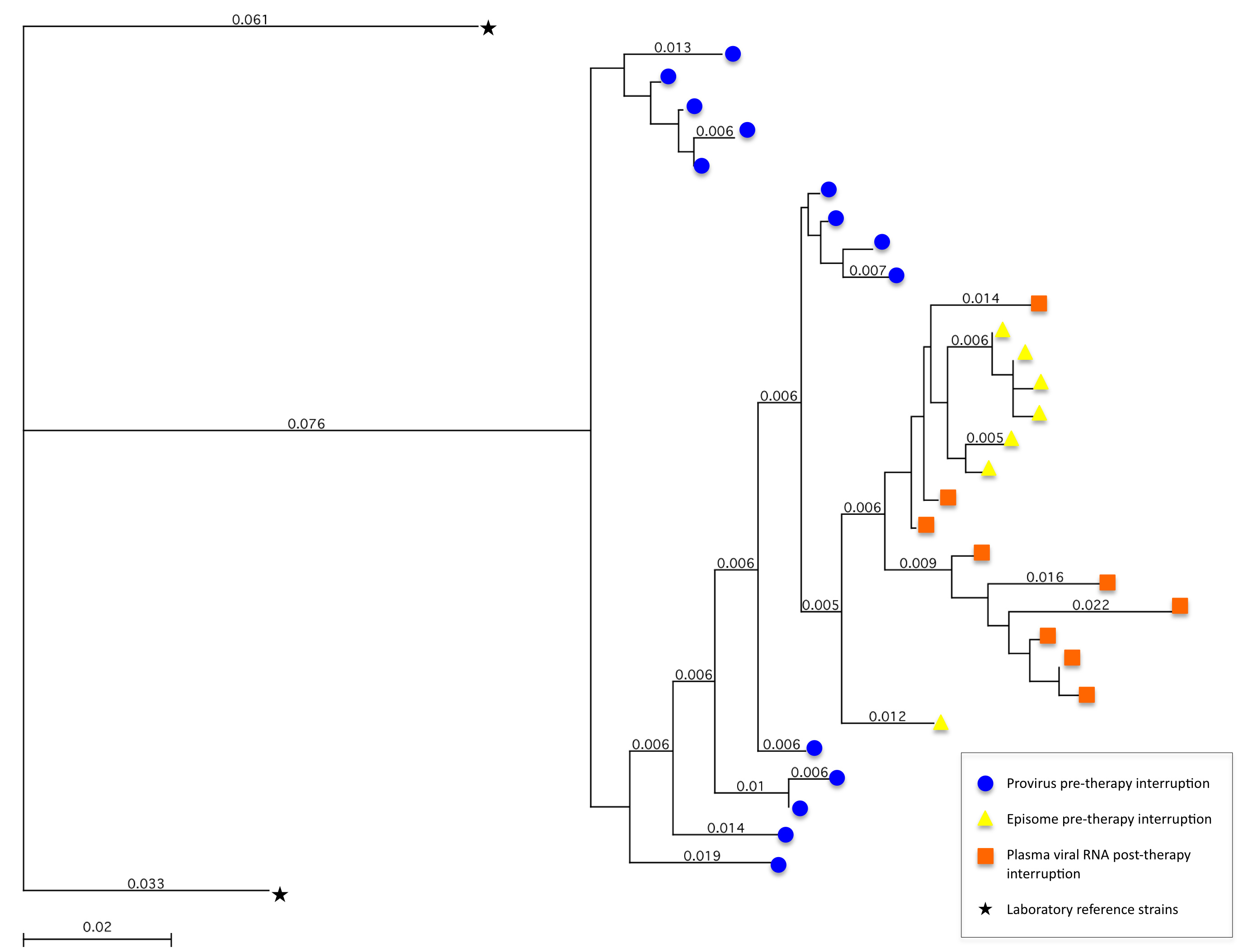

Supplement: Figure S3 — Phylogenetic tree based on the C2-V4 regions of envelope for patient 2 undergoing interruption of antiviral treatment. Phylogenetic relationships were estimated using the neighbor-joining method to generate best tree with genetic distances for episomal (yellow triangle) and proviral (blue circle) envelope sequences derived at therapy interruption to plasma viral RNA (orange square) envelope sequences obtained several weeks after rebound. Envelope sequences from HIV-1LAI and HIV-1ADA laboratory strains (black stars) are included for reference. (0.32 MB TIF) [file ppat.1001303.s003.tif]

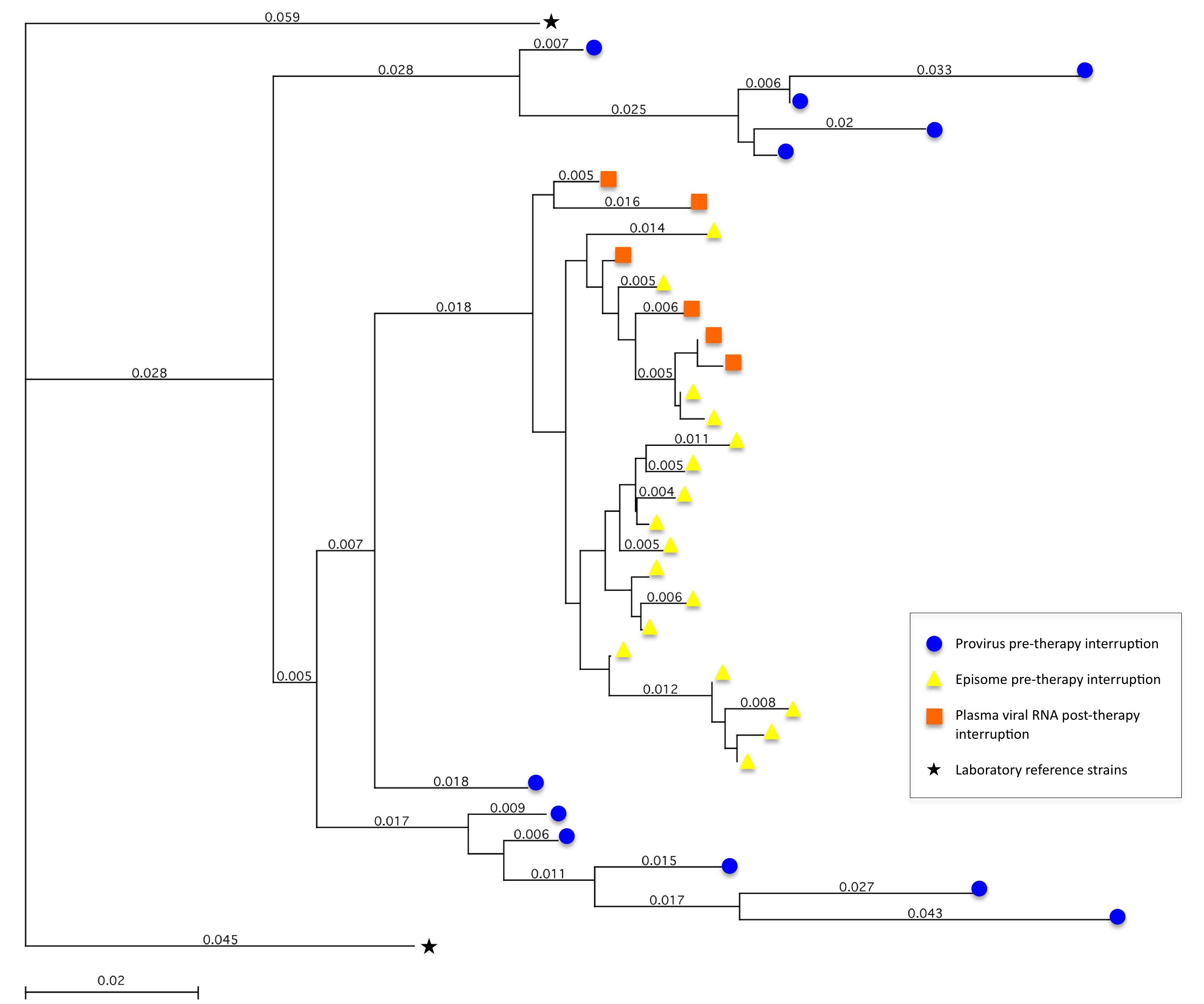

Supplement: Figure S4 — Phylogenetic tree based on the C2-V4 regions of envelope for patient 3 undergoing interruption of antiviral treatment. Phylogenetic relationships were estimated using the neighbor-joining method to generate best tree with genetic distances for episomal (yellow triangle) and proviral (blue circle) envelope sequences derived at therapy interruption to plasma viral RNA (orange square) envelope sequences obtained several weeks after rebound. Envelope sequences from HIV-1LAI and HIV-1ADA laboratory strains (black stars) are included for reference. (0.34 MB TIF) [file ppat.1001303.s004.tif]

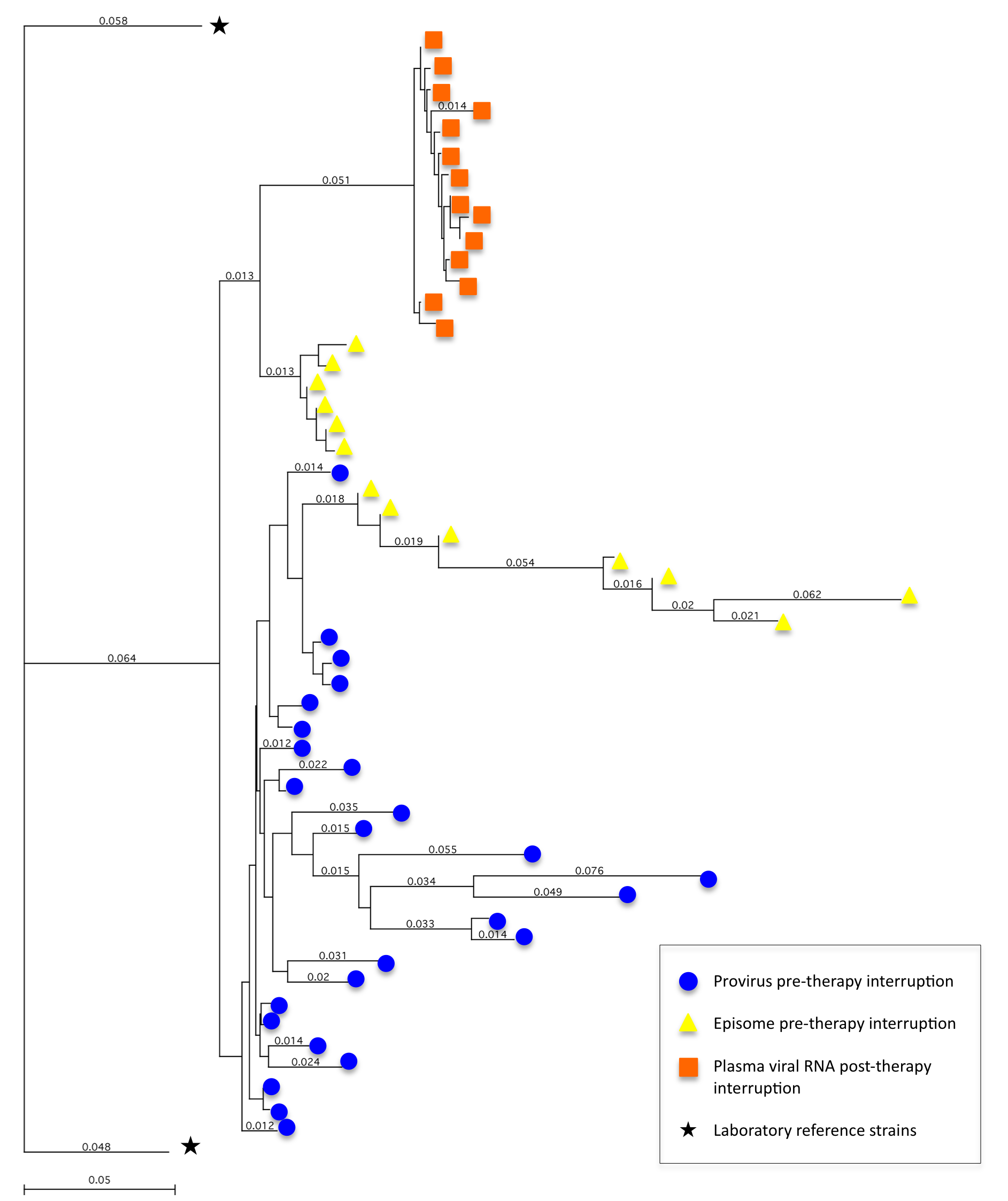

Supplement: Figure S5 — Phylogenetic tree based on the C2-V4 regions of envelope for patient 4 undergoing interruption of antiviral treatment. Phylogenetic relationships were estimated using the neighbor-joining method to generate best tree with genetic distances for episomal (yellow triangle) and proviral (blue circle) envelope sequences derived at therapy interruption to plasma viral RNA (orange square) envelope sequences obtained several weeks after rebound. Envelope sequences from HIV-1LAI and HIV-1ADA laboratory strains (black stars) are included for reference. (0.31 MB TIF) [file ppat.1001303.s005.tif]

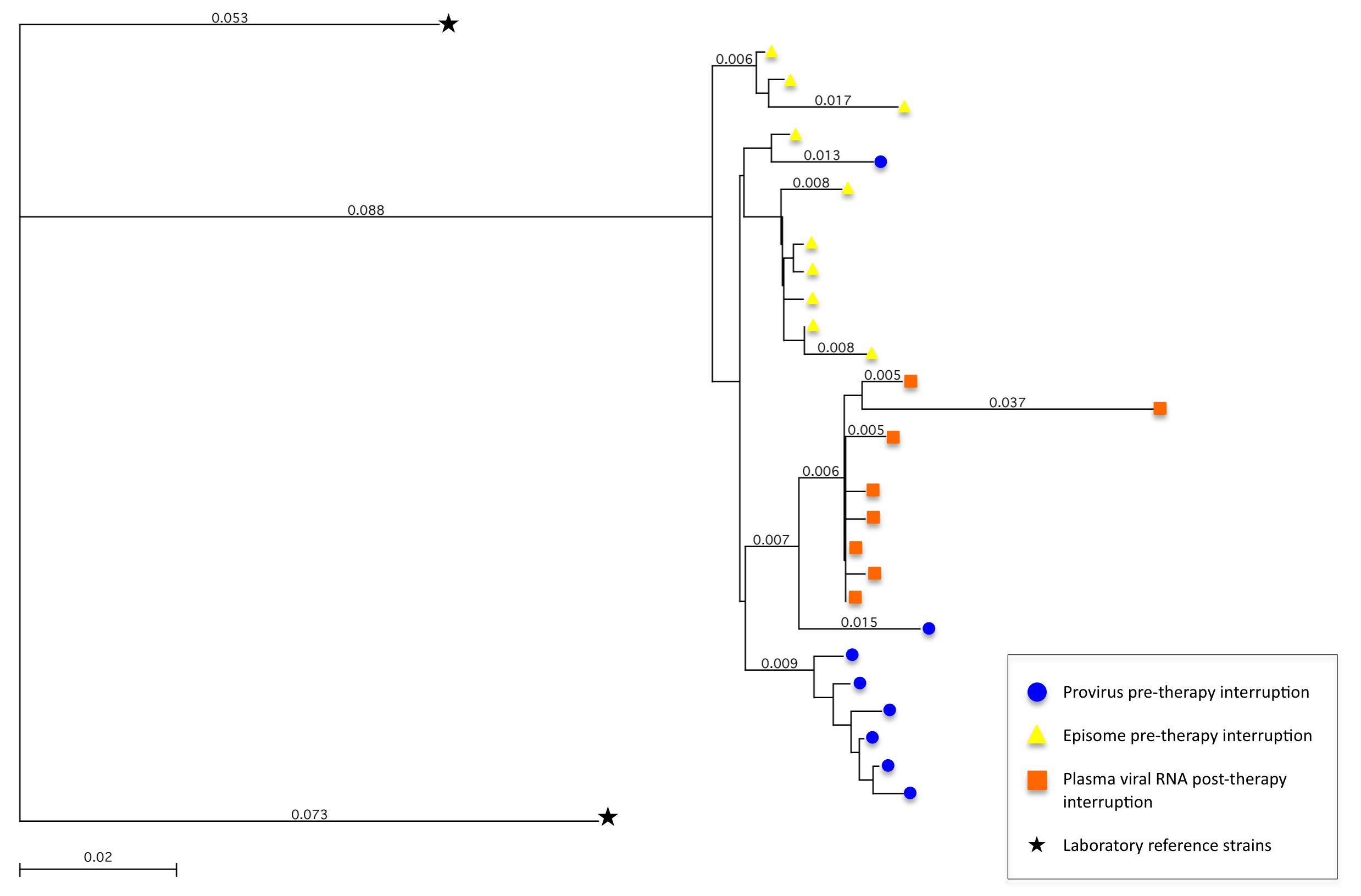

Supplement: Figure S6 — Phylogenetic tree based on the C2-V4 regions of envelope for patient 5 undergoing interruption of antiviral treatment. Phylogenetic relationships were estimated using the neighbor-joining method to generate best tree with genetic distances for episomal (yellow triangle) and proviral (blue circle) envelope sequences derived at therapy interruption to plasma viral RNA (orange square) envelope sequences obtained several weeks after rebound. Envelope sequences from HIV-1LAI and HIV-1ADA laboratory strains (black stars) are included for reference. (0.21 MB TIF) [file ppat.1001303.s006.tif]

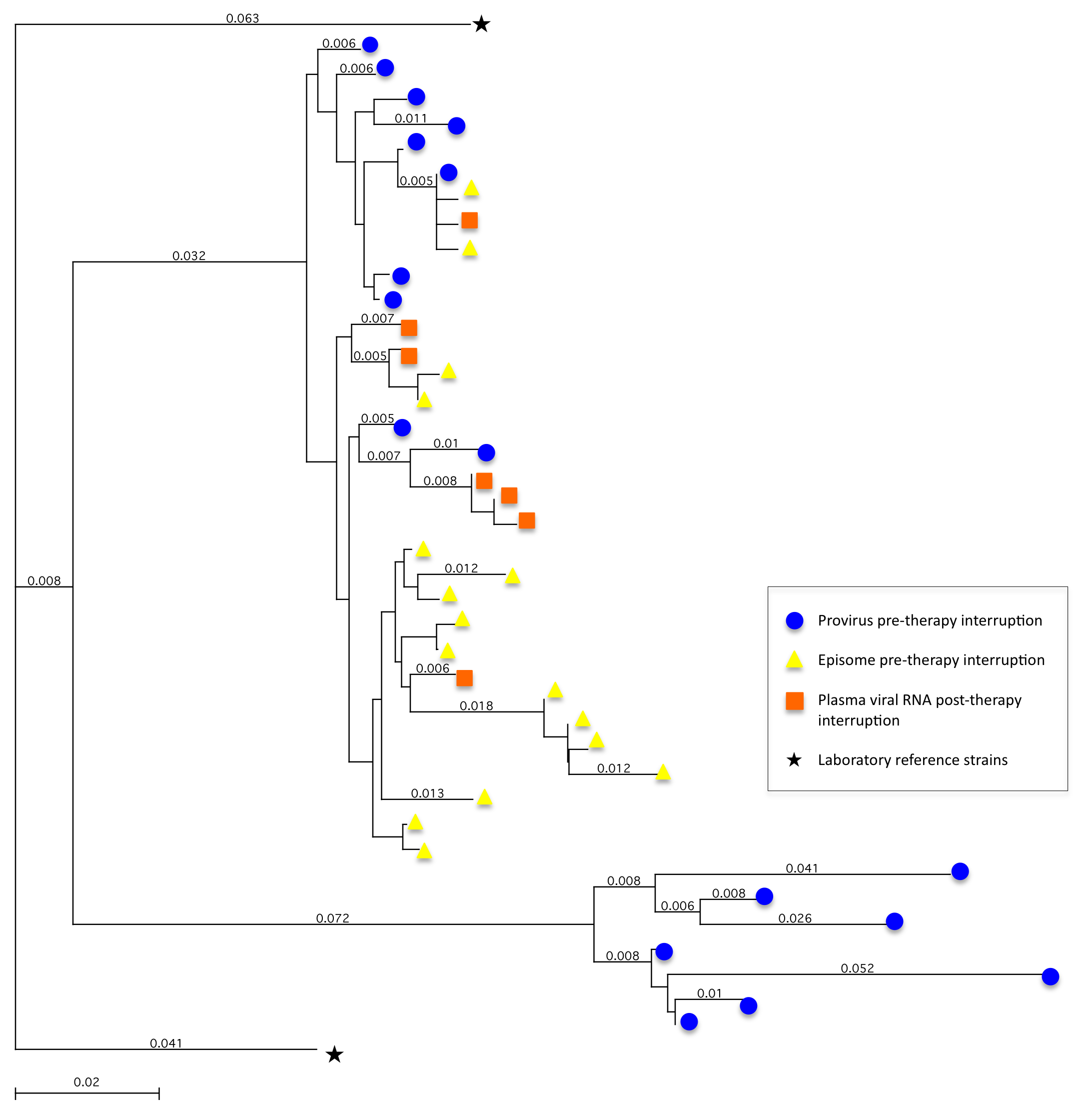

Supplement: Figure S7 — Phylogenetic tree based on the C2-V4 regions of envelope for patient 6 undergoing interruption of antiviral treatment. Phylogenetic relationships were estimated using the neighbor-joining method to generate best tree with genetic distances for episomal (yellow triangle) and proviral (blue circle) envelope sequences derived at therapy interruption to plasma viral RNA (orange square) envelope sequences obtained several weeks after rebound. Envelope sequences from HIV-1LAI and HIV-1ADA laboratory strains (black stars) are included for reference. (0.26 MB TIF) [file ppat.1001303.s007.tif]
