# Supplementary material for: Episomal Viral cDNAs Identify a Reservoir That Fuels Viral Rebound after Treatment Interruption and That Contributes to Treatment Failure
Source: PLoS Pathog. 2011 Feb 24;7(2):e1001303. doi: 10.1371/journal.ppat.1001303 (PMC3044693; doi:10.1371/journal.ppat.1001303)
Supplement: Table S1 — Tropism assignments based on HIV-1 env sequences derived from episomal cDNA and plasma viral RNA from patients with concordant results between the two assays. (0.08 MB DOC) [file ppat.1001303.s008.doc]

**Table S1.** Tropism assignments based on HIV-1 *env* sequences derived from episomal cDNA and plasma viral RNA from patients with concordant results between the two assays.

| **Patient ID and time point (# of clones)** | **log _10_ HIV-1 RNA copies/ml** | **Amplification of *env* gene from episomes** | **Phenotype Genotype*** (episomal cDNA) | **Trofile assay** (plasma viral RNA) |
| --- | --- | --- | --- | --- |
| *Patient C* |  |  |  |  |
| baseline | 4.1 | no sample | - | R5 |
| week 2 | 4.0 | NA | - | R5 |
| week 4 | 4.7 | NA | - | - |
| week 8 (5) | 4.7 | full-length | R5 | - |
| week 16 (5) | 4.4 | full-length | R5 | - |
| week 24 (4) | 4.4 | full-length | R5 | R5 |
| week 48 (3) | 4.1 | full-length | R5 | R5 |
| *Patient E* |  |  |  |  |
| baseline (5) | 4.0 | full-length | R5 | R5 |
| week 2 | 4.0 | NA | - | R5 |
| week 4 (5) | 2.4 | full-length | R5 | - |
| week 8 | 3.8 | NA | - | R5 |
| week 16 (3) | 3.7 | V3 | R5* | - |
| week 21 | 3.8 | NA | - | R5 |
| week 28 | 1.4 | NA | - | - |
| week 33 | 1.6 | NA | - | - |
| *Patient F* |  |  |  |  |
| baseline (5) | 4.1 | full-length | R5 | R5 |
| week 2 | 2.8 | NA | - | R5 |
| week 4 (5) | 3.2 | full-length | R5 | - |
| week 8 (5) | 4.9 | full-length | R5 | R5 |
| week 16 (5) | 2.5 | full-length | R5 | - |
| week 24 (5) | 2.9 | full-length | R5 | R5 |
| week 48 | 2.4 | NA | - | - |
| *Patient H* |  |  |  |  |
| baseline (5) | 4.2 | full-length | R5 | R5 |
| week 2 (5) | 4.1 | full-length | R5 | R5 |
| week 4 (5) | 3.8 | full-length | R5 | - |
| week 8 (5) | 4.9 | full-length | R5 | R5 |
| week 16 (5) | 3.6 | V3 | R5* | - |
| week 24 (5) | 3.9 | V3 | R5* | R5 |
| week 48 (5) | 3.6 | V3 | R5* | R5 |
| *Patient M* |  |  |  |  |
| baseline | 4.3 | no sample | - | R5 |
| week 2 (5) | 2.8 | full-length | R5 | R5 |
| week 4 (3) | 5.2 | full-length | R5 | - |
| week 8 (3) | 3.3 | full-length | R5 | R5 |
| week 16 (3) | 3.8 | full-length | R5 | - |
| week 23 (3) | 3.3 | full-length | R5 | R5 |
| week 33 (3) | 2.0 | full-length | R5 | - |
| week 48 (5) | 1.4 | full-length | R5 | - |
| *Patient N* |  |  |  |  |
| baseline (5) | 3.6 | full-length | R5 | R5 |
| week 2 (5) | 2.2 | full-length | R5 | R5 |
| week 4 (4) | 1.7 | full-length | R5 | - |
| week 8 | 2.0 | NA | - | R5 |
| week 16 | 2.4 | NA | - | - |
| week 24 (3) | 2.5 | full-length | R5 | - |
| week 48 (3) | 3.1 | full-length | R5 | R5 |
| *Patient P* |  |  |  |  |
| baseline | 2.5 | no sample | - | R5 |
| week 2 (2) | 1.9 | V3 | R5* | - |
| week 4 (5) | 1.8 | V3 | R5* | - |
| week 8 (5) | 4.2 | V3 | R5* | R5 |
| week 16 (2) | 3.9 | full-length | R5* | - |
| week 24 (5) | 4.3 | V3 | R5* | R5 |
| week 36 (3) | 4.4 | V3 | R5* | R5 |
| week 48 (5) | 4.5 | V3 | R5* | R5 |
|  |  |  |  |  |

NA, no amplification of full-length or short *env* fragment; DM, dual/mixed; D, dual-tropic virus

* If amplification of the full-length *env* gene was not successful or if the phenotypic assay failed, secondary information on the virus genotype was derived by amplifying and sequencing a short fragment spanning the V3 region of *env*.
