# Supplementary material for: Episomal Viral cDNAs Identify a Reservoir That Fuels Viral Rebound after Treatment Interruption and That Contributes to Treatment Failure
Source: PLoS Pathog. 2011 Feb 24;7(2):e1001303. doi: 10.1371/journal.ppat.1001303 (PMC3044693; doi:10.1371/journal.ppat.1001303)
Supplement: Table S2 — Co-receptor use at baseline as determined by the enhanced-sensitivity Trofile assay. (0.04 MB DOC) [file ppat.1001303.s009.doc]

**Table S2.** Co-receptor use at baseline as determined by the enhanced-sensitivity Trofile assay.

| **Patient ID** | **Original Trofile assay (plasma viral RNA)** | **Enhanced Trofile assay (plasma viral RNA)** | **Phenotypic assay (episomal cDNA)** |
| --- | --- | --- | --- |
|  |  |  |  |
| Patient A | DM | DM | - |
|  |  |  |  |
| Patient B | R5 | R5 | R5 |
|  |  |  |  |
| Patient C | R5 | R5 | - |
|  |  |  |  |
| Patient D | R5 | DM | - |
|  |  |  |  |
| Patient E | R5 | R5 | R5 |
|  |  |  |  |
| Patient F | R5 | R5 | R5 |
|  |  |  |  |
| Patient G | R5 | R5 | - |
|  |  |  |  |
| Patient H | R5 | R5 | R5 |
|  |  |  |  |
| Patient J | R5 | DM | X4 |
|  |  |  |  |
| Patient K | R5 | DM | - |
|  |  |  |  |
| Patient L | DM | DM | - |
|  |  |  |  |
| Patient M | R5 | R5 | - |
|  |  |  |  |
| Patient N | R5 | R5 | R5 |
|  |  |  |  |
| Patient P | R5 | R5 | - |
|  |  |  |  |

- no baseline sample available; DM, dual/mixed viral variants
